# Maresin-1 impairs cutaneous wound healing response

**DOI:** 10.1093/immhor/vlaf010

**Published:** 2025-04-02

**Authors:** Reiko Hara, Natsuko Saito-Sasaki, Yu Sawada

**Affiliations:** Department of Dermatology, University of Occupational and Environmental Health, Kitakyushu, Fukuoka, Japan; Department of Dermatology, University of Occupational and Environmental Health, Kitakyushu, Fukuoka, Japan; Department of Dermatology, University of Occupational and Environmental Health, Kitakyushu, Fukuoka, Japan

**Keywords:** maresin-1, wound healing, skin

## Abstract

Maresin-1 is a derivative of docosahexaenoic acid with strong anti-inflammatory action in various disease models. However, these effects may not always be beneficial. In instances like cutaneous diseases in which wound healing is important, inflammation is required. In this study, we investigated the effects of maresin-1 on cutaneous wound healing and found that wound healing was significantly delayed in maresin-1–treated mouse skin in the early phase of wound healing on days 1 to 3. Histological analyses revealed that maresin-1 suppressed re-epithelization in the wounded skin. Despite the direct influence of maresin-1 on keratinocyte migration, a comprehensive quantitative polymerase chain reaction analysis revealed that maresin-1–treated wound skin showed a decrease in tumor necrosis factor α, indicating that maresin-1 indirectly suppresses keratinocyte migration mediated by reduced tumor necrosis factor α derived from wounded skin, leading to delayed wound healing.

## Introduction

The skin is the outermost layer of the human body and is influenced by various external environmental factors.^1^ Once the skin surface is wounded, various physiological and pathological actions respond to environmental stimuli and drive inflammatory reactions.^2^ In addition, a defect with a large surface area in the skin is a life-threatening event due to the impairment of fundamental skin function in the human body.^3^ Therefore, the treatment of wounded skin is essential for patients.

Lifestyle greatly influences various physiological and pathological processes in the human body and plays a pivotal role in regulating inflammatory responses.^4^ Excessive inflammatory responses may cause delayed resolution of inflammation and disruption of tissue construction, leading to reduced tissue repair and function.^5^ However, an appropriate inflammatory response plays a positive role in the resolution of disease conditions, especially in wound healing.^6^ Therefore, it has been speculated that strong anti-inflammatory action may cause undesired effects in wound healing.

Omega-3 fatty acids such as docosahexaenoic acid and eicosatetraenoic acid have anti-inflammatory effects in various inflammatory diseases.^7^ In addition, derivatives of omega-3 fatty acids have been shown to have strong anti-inflammatory effects in various skin disease models.^8–11^ Among the omega-3 fatty acid derivatives, maresin-1 is a derivative of docosahexaenoic acid with strong anti-inflammatory action^12^ and is expected to show anti-inflammatory effects in cutaneous inflammatory diseases, such as psoriasis.^13^ Therefore, other inflammatory skin diseases are expected to benefit from maresin-1 treatment. In contrast, these anti-inflammatory actions might show unbeneficial effects in process requiring inflammation, such as wound healing.^14^^,^^15^ Wound healing is a classic cutaneous inflammatory response that covers surface defects of the skin, and various inflammatory cytokines play important roles in accelerating wound healing.^16^^,^^17^ Therefore, this study aimed to clarify the influence of maresin-1 on the pathogenesis of wound healing.

Recent studies have shown that maresin-1 enhances mucosal wound healing in the oral cavity.^18^ However, whether maresin-1 has beneficial effects on cutaneous wound healing remains unclear. In this study, we investigated the effects of maresin-1 on cutaneous wound healing and the possible molecular mechanisms underlying this effect.

## Materials and methods

### Reagents and mouse

Maresin-1 was purchased from Cayman Chemical and the Ki-67 antibody was purchased from Dako. The C57BL/6 mice were purchased from SLC Japan. The animal study protocol was approved by the Animal Care and Use Committee of the University of Occupational and Environmental Health and is reported in accordance with ARRIVE guidelines. All methods were performed in accordance with the relevant guidelines and regulations.

### Keratinocyte culture

FRSK cells, a spontaneously transformed keratinocyte cell line derived from rat keratinocytes, were used.^19^ Cells were seeded onto 24-well plates at a density of 1 × 10^5^ cells per well and cultured in complete Dulbecco’s Modified Eagle Medium (Nakarai Tesque) containing 10% fetal calf serum (Invitrogen), 100 U/mL penicillin, and 100 μg/mL streptomycin (Worthington Biochemical) at 37 °C in a 5% CO_2_ atmosphere.

### In vivo wound healing experiment

A 6-mm full-thickness excisional wound was created on the shaved dorsal skin surface of 8-wk-old female C57BL/6 wild-type mice under sterile conditions. Wounds were treated topically every 24 h for 7 d with 100 ng maresin-1 or ethanol vehicle control. The wound area was measured using ImageJ software, version 1.48 (National Institutes of Health).

### Polymerase chain reaction

Total RNA was extracted from the keratinocytes using the PureLink RNA Mini Kit (Thermo Fisher Scientific). Purified total RNA was reverse-transcribed into complementary DNA (cDNA) with first-strand cDNA synthesis kit for real-time polymerase chain reaction (PCR) (Verso cDNA synthesis kit; Thermo Fisher Scientific). Real-time quantitative PCR was performed by monitoring the synthesis of dsDNA during PCR cycles using PowerUp SYBR Green Master Mix (Thermo Fisher Scientific) with the ABI Prism 7000 sequence detection system (Applied Biosystems). Gene expression levels were quantified using quantitative PCR and normalized to GAPDH. The results are presented as Relative Expression (target gene/GAPDH). The primer sequences used for quantitative PCR are shown in Table S1.

### Histological analysis

The mouse back skin was excised, fixed in 10% formaldehyde, and embedded in paraffin. Sections of 5-μm thickness were prepared and stained with hematoxylin and eosin. To minimize the potential variability in wound sectioning caused by the irregular shape of wounds, we consistently bisected the ulcer along its longest axis and fixed the samples in formalin, ensuring uniform sectioning across all specimens. Wound length and epithelialization were calculated using the image viewer software VS-OlyVIA VS-ASW (version 2.9.2; Olympus).

### Immunostaining for Ki-67

Monoclonal antibodies against Ki-67 (Dako) were used to calculate Ki-67 index. The slices were placed in 0.01 M citrate buffer (pH 6.0), heated in an autoclave at 120 °C for 5 min and immunostained by incubating with the primary antibody for 2 h at room temperature. The EnVision+ Dual-link system from Dako Cytomation uses diaminobenzidine as the chromogen to observe antibody binding. Five high-power fields in the epidermis of each slice (total approximately 400) were used to calculate the number of positive Ki-67 keratinocytes. The number of positive Ki-67 cells were calculated using VS-OlyVIA VS-ASW.

### In vitro scratch assay

Rat keratinocytes (3 × 10^5^ cells/well) were seeded onto 6-well culture plates and incubated in a CO_2_ incubator. The scratch was made using a P1000 pipette tip, and the “area” measurement refers to the surface area observed within a single microscopic field. The wounded cells were washed twice with culture medium to remove the detached cells and treated with media containing 10 nM maresin-1 or vehicle. Images of the wounds were automatically acquired in the CO_2_ incubator using a Bio Zero BZ-800 (Keyence), and the closure area was measured. For time-lapse imaging, typical kinetic updates were taken at 5 min intervals for 13 h. The data were analyzed using the ImageJ software.

### Statistical analysis

All statistical analyses were performed using the GraphPad Prism software version 9.3.1 (GraphPad Software). One-way analysis of variance or Student’s *t* test was used to calculate the statistical differences. Statistical significance was set at *P* < 0.05.

## Results

### Maresin-1 impaired cutaneous wound healing

**Figure 1.**
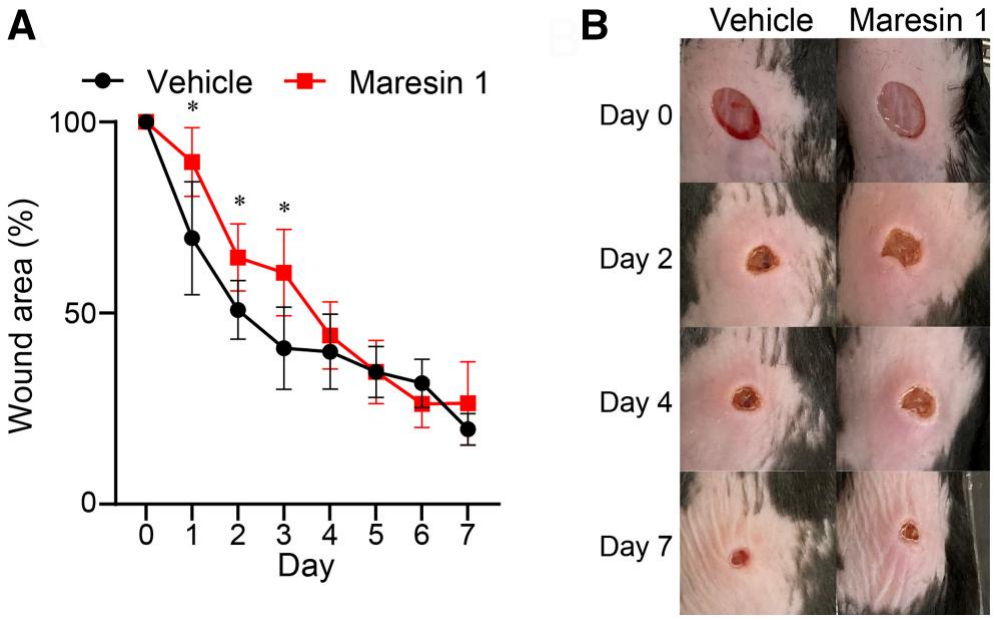
Wound healing measurement and manifestation images. (A) Wound healing was evaluated by measuring the wounded area with or without topical maresin-1 applied daily on the wounded skin in vivo (n = 5). (B) Cutaneous wound closure with vehicle or maresin-1 treatment. Statistical analysis was performed using Student’s *t* test, with a significance threshold set at *P* < 0.05. Asterisks indicate statistically significant differences. *P < 0.05.

Based on a previous study showing that maresin-1 accelerates oral mucosal would healing,^18^ we expected the same effect on skin wound healing. Many studies have elucidated the strong anti-inflammatory actions of maresin-1 in various inflammatory diseases. However, as the inflammatory response in wound healing repair is essential, strong anti-inflammatory action in wounded skin might cause delayed wound repair. To clarify this, topical maresin-1 was applied daily to wounded skin in vivo, and the influence of maresin-1 on cutaneous wound healing was evaluated by measuring the wound area in the presence or absence of maresin-1. Unexpectedly, wound healing was significantly delayed in maresin-1–treated mouse skin compared to vehicle-treated mice (Fig. 1A, B). Significant differences were observed in mice treated with maresin-1 during the early phase of wound healing on days 1 to 3. These unexpected results prompted us to investigate the role of maresin-1 in the pathogenesis of delayed wound healing.

### Re-epithelization was impaired in maresin-1–treated mouse

**Figure 2.**
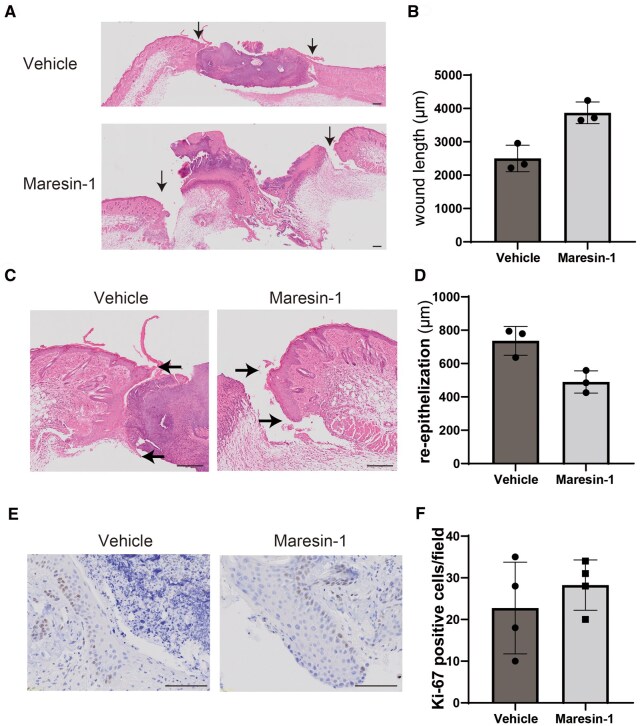
Histological evaluation in wound healing. (A) Hematoxylin and eosin staining of the wounded skin in which wound length was measured using histological analysis. Arrows indicate the edge of the wounded skin to highlight the length of wound area. (B) The difference in wound length in presence of vehicle and maresin-1 treatment (n = 3). (C) Hematoxylin and eosin staining of epithelized skin. Arrows indicated the edge of the re-epithelized skin to highlight the advancement of re-epithelization length. (D) Re-epithelized skin was measured, and the difference in the presence of vehicle and maresin-1 treatment is shown (n = 3). (E) Ki-67 immunostaining to evaluate the keratinocyte proliferation in vivo skin in the presence of vehicle or maresin-1 treatment. (F) The positive cells were evaluated and the difference of the positive Ki-67 cells is shown (n = 4). The distance (mm) was directly calculated by the software. Scale bars = 200 μm (A) and 100μm (C, E).

Because we observed that maresin-1 delayed wound healing in the skin, we determined the differences in histological characteristics between the vehicle and maresin-1 treatments. A skin sample taken on day 3 showed significantly impaired wound length, as determined by measuring the lengths between the wound margins (Fig. 2A, B, middle). Re-epithelialization was determined by measuring the lengths of the wounds within the epidermis of maresin-1–treated mice (Fig. 2C, D), suggesting that maresin-1 might suppress epithelialization in the wound healing process.

We evaluated the influence on keratinocyte proliferation in maresin-1 treatment as a possible reason for delayed wound healing in maresin-1–treated mice. However, keratinocyte proliferation, evaluated by counting Ki67-positive cells, was unaffected by maresin-1 treatment, suggesting that maresin-1 did not suppress keratinocyte proliferation (Fig. 2E, F). This indicates other possible influences of maresin-1 on keratinocytes to suppress re-epithelization in wounded skin.

### No direct influence of maresin-1 in keratinocyte migration

**Figure 3.**
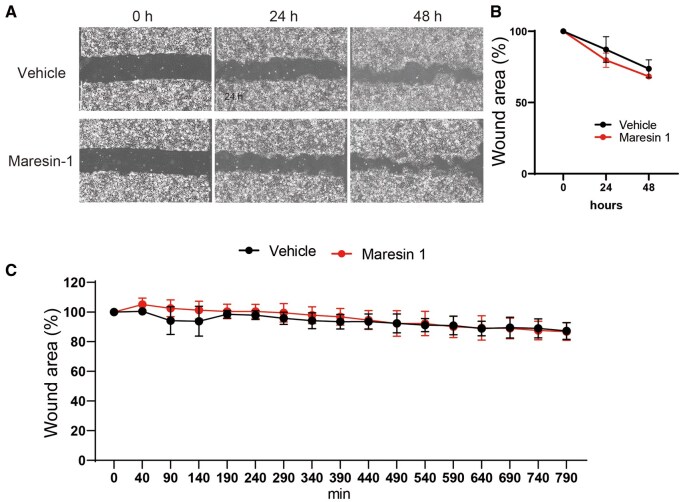
The evaluation of keratinocyte migration in vitro. (A) In vitro scratch assay images. The scratch assay was performed using a P1000 pipette tip. The wound area was measured within a single microscopic field. (B) The effect of maresin-1 was measured using an in vitro scratch assay at 24 and 48 h to evaluate the outcome of the wound area. (C) Early-phase keratinocyte migration was evaluated using time-lapse imaging for 13 h to determine the influence of maresin-1 in vitro. Statistical analysis was conducted using Student’s *t* test, with a significance threshold set at *P* < 0.05. The number of scratch replicates was n = 3.

Maresin-1 suppresses the re-epithelization of wounded skin. Therefore, we hypothesized that maresin-1 negatively regulates keratinocyte migration and delays wound healing. To examine the direct role of maresin-1 in keratinocyte migration during wound repair, we conducted an in vitro scratch assay using rat keratinocytes under in vitro maresin-1 treatment, as described previously.^13^ We first examined the influence of maresin-1 on keratinocyte migration 24 and 48 h after starting the experiment in the presence and absence of maresin-1. However, there was no significant difference in the wound closure rate between the vehicle and maresin-1 groups at 24 and 48 h (Fig. 3A, B). Because we could not exclude the possibility of a role of maresin-1 in keratinocyte migration during the early phase, we observed keratinocyte migration for 13 h to reveal the influence of maresin-1. However, the wound closure rate was similar in the treatment with vehicle and maresin-1 during the early phase, up to 13 h (Fig. 3C; Videos S1 and S2). These findings suggest that maresin-1 exhibits no direct action on keratinocyte migration *in vitro* and may indirectly influence delayed re-epithelization in wound healing.

### Maresin-1 suppressed inflammatory cytokine profiles in the lesional skin

**Figure 4.**
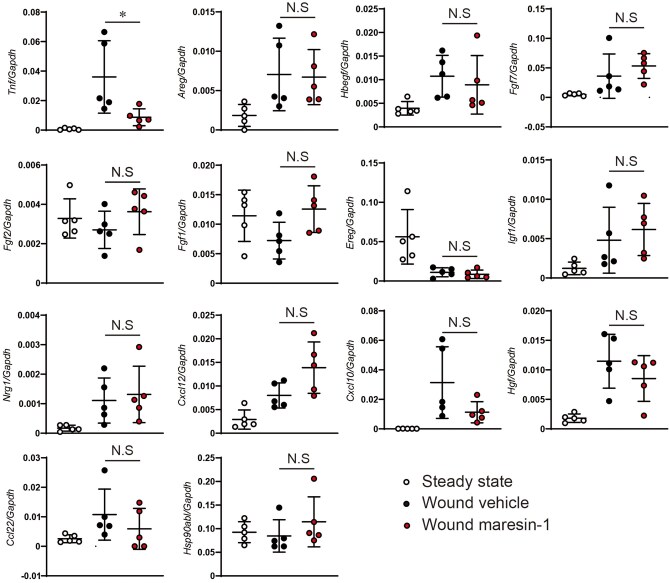
Quantitative PCR evaluation. Representative factor genes for the regulation of keratinocyte migration were evaluated by quantitative PCR in the wounded skin 24 h after the start of the wound healing experiment. N.S, not significant. *P < 0.05.

Because maresin-1 did not directly influence keratinocyte migration, we speculated that maresin-1 might indirectly regulate keratinocyte migration. It is possible that maresin-1 suppresses inflammatory cytokine and chemokine production. Therefore, we hypothesized that maresin-1 might suppress the interaction with keratinocytes mediated by the suppression of cytokine production by other cells in the skin. Keratinocyte migration is influenced by various inflammatory cytokines and chemokines that accelerate wound repair.^20^ Because maresin-1 suppresses inflammatory cytokine production in various inflammatory disease models, we speculated that maresin-1 may contribute to the suppression of beneficial inflammatory cytokine production in lesional skin, leading to delayed wound repair. To assess this possibility in mice treated with maresin-1, we collected the wounded skin 1 d after treatment and examined the gene expression of these inflammatory cytokines and chemokines. Although various factors are involved in the regulation of keratinocyte migration, we noticed a significant increase in *Tnf* expression after skin injury (Fig. 4). Because tumor necrosis factor α (TNF-α) is essential for wound healing and promotion of keratinocyte migration to accelerate re-epithelization in the skin,^21^^,^^22^ decreased the cytokine production by maresin-1 treatment was thought to contribute to delayed wound healing.

## Discussion

Our study showed that maresin-1 impaired the production of a cytokine, TNF-α, that is essential for keratinocyte migration, leading to delayed wound repair in the skin. Although maresin-1 has shown various beneficial impacts on inflammatory disease and malignancies, we should keep in mind that the anti-inflammatory action of maresin-1 might worsen the disease by suppressing wound healing.

A previous study has shown the opposite action of maresin-1 in mucosal wound healing. Although the repair process of oral mucosal wounds is similar to that of skin wounds,^23^ the oral mucosa shows rapid and minimal scar formation.^23^ Oral wounds showed a lower inflammatory response with decreased infiltration of inflammatory cells and inflammatory cytokines.^24^^,^^25^ Although wound repair in the mucosa is not affected by the anti-inflammatory action of maresin-1, certain inflammatory cytokines play a crucial role in skin wound healing. Maresin-1 does not directly inhibit oral keratinocyte proliferation; oral human keratinocytes reduce proliferation mediated by TNF-α,^18^ which is essential for skin keratinocyte migration. Therefore, the anti-inflammatory action of maresin-1 is disadvantageous for skin wound healing.

Inflammatory cytokines enhance skin keratinocyte migration as TNF-α is produced by both macrophages and neutrophils.^26^ TNF-α indirectly enhances collagen deposition, which is blocked by maresin-1.^27^ Consistently, TNF-α deficiency impairs matrix metalloproteinases, such as matrix metalloproteinase-9, which is essential for keratinocyte migration.^21^ Matrix metalloproteinase-9 production in keratinocytes is enhanced by interleukin-1β, transforming growth factor β1, and TNF-α.^28^ These findings suggest that maresin-1 indirectly regulates keratinocyte migration by inhibiting key inflammatory cytokines. Previous studies have demonstrated the anti-inflammatory action of maresin-1 in suppressing various inflammatory cytokines.^12^ Maresin-1 suppresses TNF-α production by macrophages,^29^ suggesting that maresin-1 can impair wound healing mediated by macrophage-secreted TNF-α in wounded skin. Consistently, DHA-supplemented mice showed impaired wound healing by the suppression of inflammatory cytokines, especially TNF-α.^30^

Due to its strong anti-inflammatory action, maresin-1 may be disadvantageous for clinical applications. For example, cutaneous infectious diseases might show impaired antimicrobial action on the skin. Maresin-1 reduces interleukin-17 production in the skin,^13^ which positively drives anti-microbial peptide production such as HBD-2 and cathelicidin for bactericidal actions.^31^^,^^32^ These findings provide us with a careful approach for specific inflammatory skin diseases.

Early wound contraction plays a significant role in murine wound closure, particularly within the first few days.^33^ In this study, the most pronounced difference in wound area was observed between days 0 and 2, suggesting that maresin-1 may have influenced both contraction and re-epithelialization. Although we did not specifically assess wound contraction, its potential contribution cannot be excluded.

Our study does not directly assess inflammatory cell dynamics or alternative sources of TNF-α within the wound environment. The early differences in TNF expression and keratinocyte migration observed in our study suggest the involvement of other cell types. Fibroblasts, which play a key role in keratinocyte cross-talk during wound healing, are a potential early source of TNF-α.^34^ Additionally, we acknowledge that we have not directly provided evidence for a deficiency in TNF-α protein or its causal role in the transient decrease in healing.

Our findings suggest that the potent anti-inflammatory effects of maresin-1 may impair the early phases of wound healing by suppressing the necessary inflammatory response. While early inflammation is essential for initiating the wound healing process, its resolution is also critical for the transition to the proliferative phase. The precise timing of maresin-1 administration could therefore be a key factor in determining its impact on wound repair. A delayed administration of maresin-1 initiated after the acute inflammatory phase might allow for the necessary early inflammatory response while still promoting resolution and tissue repair. Additionally, maresin-1 may be particularly beneficial in models of chronic wounds characterized by persistent inflammation, such as diabetic wounds, in which excessive and prolonged inflammation hinders proper healing.

The species difference between the scratch assay, which utilizes rat-derived cells, and the in vivo wound healing model in mice should be considered when interpreting the results. Species-specific variations in cellular responses, inflammatory mediators, and tissue repair mechanisms may influence the wound healing process.

Thus, maresin-1 is a potential therapeutic candidate for the treatment of inflammatory diseases. In contrast, maresin-1 exhibits opposite pharmacological effects under certain conditions, such as in wound repair. Therefore, we should keep in mind that maresin-1 is not universal, but it does show strong potential as a treatment for human diseases.

## Author contributions

R.T.-N., N.S.-S., and Y.S. contributed equally to this study. R.T.-N., N.S.-S., and Y.S. conceptualized, designed the study, and performed data collection and analysis. Y.S. contributed to the interpretation of the results and manuscript drafting. All authors critically revised the manuscript, approved the final version, and agreed to be accountable for all aspects of the work.

## Supplementary Material

vlaf010_Supplementary_Data

## Data Availability

The datasets used and/or analyzed during the current study available from the corresponding author on reasonable request.
