# Supplementary material for: Maresin-1 impairs cutaneous wound healing response
Source: Immunohorizons. 2025 Apr 2;9(5):vlaf010. doi: 10.1093/immhor/vlaf010 (PMC11964490; doi:10.1093/immhor/vlaf010)
Supplement: vlaf010_Supplementary_Data [file vlaf010_supplementary_data.zip › IMMHOR-24-00088-s04.pdf]

Supplementary movie 1. Time-lapse imaging of keratinocyte migration under vehicle treatment

Time-lapse imaging of *in vitro* scratch assay was performed in the presence of vehicle for 13 hours *in vitro*.

Supplementary movie 2. Time-lapse imaging of keratinocyte migration under maresin-1 treatment

Time-lapse imaging of *in vitro* scratch assay was performed in the presence of maresin-1 for 13 hours *in vitro*.
