# Supplementary material for: Maresin-1 impairs cutaneous wound healing response
Source: Immunohorizons. 2025 Apr 2;9(5):vlaf010. doi: 10.1093/immhor/vlaf010 (PMC11964490; doi:10.1093/immhor/vlaf010)
Supplement: vlaf010_Supplementary_Data [file vlaf010_supplementary_data.zip › IMMHOR-24-00088-s03.pdf]

Supplementary Table 1. Primer Sequences Used for qPCR Analysis

mouse *Gapdh* Forward   AGGTCGGTGTGAACGGATTTG

mouse *Gapdh* Reverse : TGTAGACCATGTAGTTGAGGTCA

mouse *Tnf* Forward : CCCTCACACTCAGATCATCTTCT

mouse *Tnf* Reverse : GCTACGACGTGGGCTACAG

mouse *Areg* forward: GGT CTT AGG CTC AGG CCA TTA

mouse *Areg* reverse: CGC TTA TGG TGG AAA CCT CTC

mouse *Hbegf* forward : CGG GGA GTG CAG ATA CCT G

mouse *Hbegf* reverse : TTC TCC ACT GGT AGA GTC AGC

mouse *Fgf1* forward : CCC TGA CCG AGA GGT TCA AC

mouse *Fgf1* reverse: GTC CCT TGT CCC ATC CAC G

mouse *Fgf2* forward: GCG ACC CAC ACG TCA AAC TA

mouse *Fgf2* reverse: TCC CTT GAT AGA CAC AAC TCC TC

mouse *Fgf7* forward : CTC TAC AGG TCA TGC TTC CAC C

mouse *Fgf7* reverse : ACA GAA CAG TCT TCT CAC CCT

mouse *Ereg* forward : CTG CCT CTT GGG TCT TGA CG

mouse *Ereg* reverse: GCG GTA CAG TTA TCC TCG GAT TC

mouse *Igf1* forward : CTG GAC CAG AGA CCC TTT GC

mouse *Igf1* reverse: GGA CGG GGA CTT CTG AGT CTT

mouse *Nrg1* forward: ATG GAG ATT TAT CCC CCA GAC A

mouse *Nrg1* reverse : GTT GAG GCA CCC TCT GAG AC

mouse *Cxcl10* forward : CCA AGT GCT GCC GTC ATT TTC

mouse *Cxcl10* reverse : GGC TCG CAG GGA TGA TTT CAA

mouse *Cxcl12* forward : TGC ATC AGT GAC GGT AAA CCA

mouse *Cxcl12* reverse: TTC TTC AGC CGT GCA ACA ATC

mouse *Hgf* forward: ATG TGG GGG ACC AAA CTT CTG

mouse *Hgf* reverse : GGA TGG CGA CAT GAA GCA G

mouse *Ccl22* forward : AGG TCC CTA TGG TGC CAA TGT

mouse *Ccl22* reverse : CGG CAG GAT TTT GAG GTC CA

mouse *Hsp90ab1* forward : GTC CGC CGT GTG TTC ATC AT

mouse *Hsp90ab1* reverse : GCA CTT CTT GAC GAT GTT CTT GC

Table 1 legend

The primer sequences listed were used for quantitative PCR (qPCR) analysis. These primers were designed to amplify specific genes in mouse samples.
